# Multi-omics identifies a T2DM-associated immune-regulatory network modulated by electroacupuncture

**DOI:** 10.3389/fendo.2026.1742131

**Published:** 2026-04-01

**Authors:** Chuyun Chen, Wengai Huang, Lihong Lu, Yongsi Liu, Qin Zhang, Yalin She, Xiaoting Huang, Yingmin Deng, Liming Lu

**Affiliations:** 1 Medical College of Acu-Moxi and Rehabilitation, Guangzhou University of Chinese Medicine, Guangzhou, China; 2 The Affiliated Traditional Chinese Medicine Hospital, Guangzhou Medical University, Guangzhou, China; 3 Guangzhou University of Chinese Medicine, Guangzhou, China; 4 Clinical Research and Big Data Laboratory, Research Center for Acupuncture and Moxibustion, Medical College of Acu-Moxi and Rehabilitation, Guangzhou University of Chinese Medicine, Guangzhou, China

**Keywords:** colocalization analysis, immune, mendelian randomization, multi-omics, type 2 diabetes

## Abstract

**Background:**

Recent studies suggest a link between immune-related processes and type 2 diabetes (T2DM), yet the specific genes and mechanisms remain unclear. This study aimed to explore the mechanistic relationship by integrating multi-omics data.

**Methods:**

We identified T2DM-associated candidate genes through summary data-based Mendelian Randomization (SMR) and colocalization analyses using immune-related QTL data and large-scale GWAS data. We then functionally validated key genes in a high-fat diet-induced T2DM mouse model, assessing gene expression and the therapeutic effects of electroacupuncture (EA) using RT-qPCR.

**Results:**

SMR and colocalization analyses identified *PLXNB2* and *LTBP3* as core candidate genes associated with T2DM. In the T2DM mouse model, we not only confirmed the abnormal expression of Plxnb2 and Ltbp3 but also observed coordinated changes in key upstream/downstream molecules, such as *TGF-β*, revealing a functional immune regulatory network. Importantly, EA intervention significantly modulated the expression of the entire network, including *Plxnb2*, *Ltbp3*, and *TGF-β*, confirming their functional relevance.

**Conclusions:**

Integrating large-scale human genetic data with *in vivo* validation, our study highlights the significance of immune-related genes—particularly *LTBP3*, *PLXNB2*, *TGF-β*, and *IgA*—in T2DM pathogenesis. The modulation of these genes by EA treatment provides direct evidence for the molecular mechanisms underlying its therapeutic effect on T2DM.

## 1 Introduction

Type 2 diabetes mellitus (T2DM) is a widespread metabolic disorder, primarily driven by obesity, affecting around 10% of adults globally (1). It involves insulin resistance and β-cell dysfunction, leading to hyperglycemia and complications such as cardiovascular disease, nephropathy, and neuropathy (2). Treatments include lifestyle changes, medications, and bariatric surgery, yet optimal glycemic control remains difficult (3).Recent studies highlight immune dysregulation as a key factor in T2DM (4), and this immune-metabolic link suggests new therapeutic avenues.

Recent research has increasingly recognized the critical role of immune dysregulation in the pathogenesis of various diseases. Chronic low-grade inflammation, frequently associated with obesity, is a key driver of both insulin resistance and β-cell failure, which are central to the progression of T2DM (4). Immune cells, particularly macrophages, infiltrate metabolic tissues such as adipose tissue, liver, and pancreas, triggering an inflammatory response that disrupts insulin signaling pathways and contributes to β-cell apoptosis (5). This inflammation links obesity to T2DM and underscores the immune system’s involvement in disease progression (6). The systemic immune-inflammation index (SII) has emerged as a significant marker associated with complications of T2DM, such as diabetic kidney disease (DKD), further highlighting the importance of immune responses in the disease’s pathology (7). These insights suggest that targeting immune pathways could provide new therapeutic opportunities for managing T2DM and its associated complications.

Mendelian Randomization (MR) is a powerful epidemiological tool that uses genetic variations to infer causal relationships between risk factors and disease outcomes, providing valuable insights into the potential interactions between immune-related genes and T2DM (8). However, traditional MR studies often rely on single-omics data, which may not fully capture the multidimensional biological mechanisms underlying complex diseases. Multi-omics analysis enhances the accuracy and reliability of causal inference by integrating data from genomics, transcriptomics, epigenomics, and proteomics (9). In this study, we applied summary data-based Mendelian Randomization (SMR) analysis, integrating multi-omics data, including gene expression, DNA methylation, and protein expression, to identify and validate genetic variants associated with T2DM (10). Then we induced obese T2DM mouse model and executed targeted *in vivo* functional assays that directly corroborated the prioritized immune gene candidates. This approach not only offers a more comprehensive perspective by exploring the relationships between immune-related genes and T2DM across multiple biological layers, but also bridges the persistent gap between genomic epidemiology and therapeutic target prioritization, laying a robust foundation for precision medicine in T2DM.

This study utilizes SMR to identify immune-related pathogenic genes in T2DM. By integrating multi-omics data, we aim to uncover genetic variants associated with T2DM risk, providing preliminary and robust evidence. This work seeks to offer new insights into the complex genetic landscape of T2DM and to lay the theoretical foundation for developing targeted therapies and personalized medical strategies.

## 2 Methods

### 2.1 Bioinformatic and multi-omics analysis

#### 2.1.1 Study design

**Figure 1:**
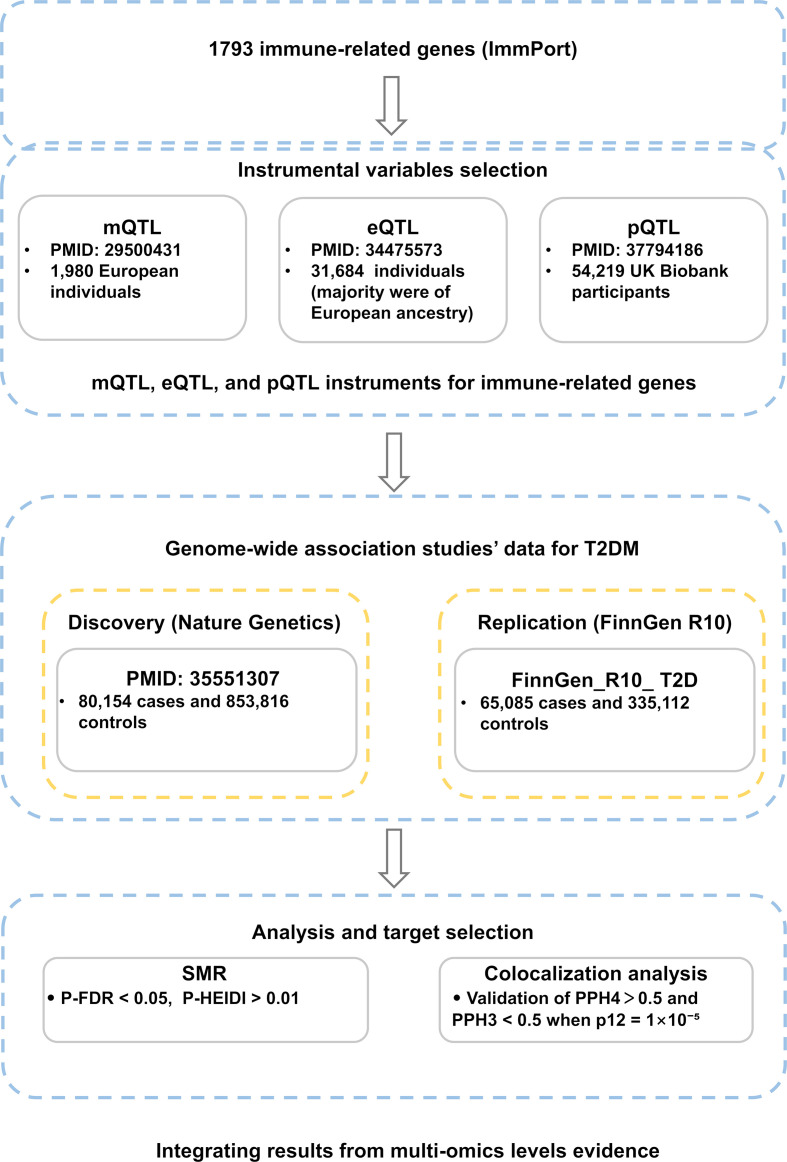
Flowchart of the study design and multi-omics Mendelian randomization analyses.

In this study, immune-related genes were extracted as instrumental variables at three levels: methylation, gene expression, and protein abundance. Independent MR analyses were performed at each biological level to investigate their relationship with T2DM. GWAS data from Nature Genetics was used as the primary discovery dataset (11), and T2DM GWAS data from the FinnGen R10 database was used for validation. There was no sample overlap between the exposure and outcome groups. To strengthen causal inference, colocalization analysis was also performed. By integrating the results of these three distinct MR analyses, we identified causal candidate genes and conducted further tissue-specific validation. **Figure 1** summarizes the study design and workflow for genetic variant selection and analytical methods.

#### 2.1.2 Data resources

We downloaded immune-related genes from the ImmPort database, resulting in 1, 793 unique genes after removing duplicates. Summary data on blood mQTL was obtained from a meta-analysis of two cohort studies: the Brisbane Systems Genetics Study (n = 614) and the Lothian Birth Cohort (n = 1, 366) (12). Blood eQTL data were sourced from the eQTLGen Consortium, covering blood gene expression data from 31, 684 individuals. Summary data on blood protein quantitative trait loci (pQTL) was derived from the UKB-PPP cohort by Benjamin et al., exploring the proteome-genome intersection in human disease, with data from 54, 219 donors.

Summary statistics for the T2DM GWAS were obtained from a genome-wide association study in Nature Genetics, which included multiple ethnic groups with 80, 154 cases and 853, 816 controls. In the validation phase, we used the FinnGen dataset, which included 65, 085 cases and 335, 112 controls.

To assess tissue-specific expression of target genes, we retrieved tissue-specific eQTL data from the GTEx database (https://gtexportal.org/home/), exploring potential causal effects of genes on T2DM. The GTEx v8 dataset includes 838 donors and 17, 382 samples from 52 tissues and two cell lines. For T2DM-related analysis, we used eQTL data from pancreatic tissue, as the pancreas plays a key role in insulin production and glucose regulation, making it crucial for understanding the pathogenesis of T2DM.

All summary statistics used for MR analysis were derived from previously published studies (detailed information including the predominantly European ancestry of these cohorts is provided in **Supplementary Table 1**), all of which had received ethical approval.

#### 2.1.3 Summary DATA-BASED MR ANALYSIS

We used SMR to estimate the relationship between immune-related gene methylation, expression, and protein abundance with T2DM. SMR can achieve higher statistical power than traditional MR analysis based on the most relevant cis-QTLs when the exposure and outcome are derived from two independent cohorts with large sample sizes (13). In this study, the most significant cis-QTL was selected using a window centered around the corresponding gene (± 1000 kb) and a P-value threshold of 5.0 × 10^-8^ (14). SNPs with allele frequency differences exceeding the specified threshold (set to 0.2 in this study) in any pairwise datasets (including LD reference samples, QTL summary data, and outcome summary data) were excluded. For eQTL, mQTL, and pQTL, the default setting for the allowed allele frequency difference was 0.05.

In addition to SMR analysis, we employed a multi-SNP SMR analysis method, integrating mQTL, eQTL, and pQTL data to investigate causal relationships between DNA methylation and gene expression, as well as between gene expression and protein abundance (13). SMR analysis treated mQTL as the exposure and eQTL as the outcome, or eQTL as the exposure and pQTL as the outcome. These analyses aimed to discuss whether the expression of target genes is regulated by methylation at specific CpG sites within their functional regions or to validate whether the expression of target genes regulates the abundance of their encoded proteins. The study focused on the results obtained using this method.

P-values from the SMR analysis were corrected using the Benjamini-Hochberg method, and the BH-adjusted P-values were used to assess the significance of the results. Subsequently, results without pleiotropy (P > 0.01 in the HEIDI test) were selected. Specifically, in cases where the BH-adjusted SMR P-value < 0.05, and the results met the criteria of P FDR < 0.05 and P HEIDI > 0.01, these results were used for subsequent eQTL, mQTL, and pQTL colocalization and integration analysis (15). The results from the integration analysis were further validated using tissue-specific eQTL data and analyses from the discovery dataset.

SMR and HEIDI tests were implemented using the SMR software tool (version 1.3.1). All statistical analyses were conducted using R (v4.3.0). The R packages “ggplot2” and “ggrepel” were used for Manhattan plots, while “forestplot” was used for forest plots. SMRLocusPlot and SMREffectPlot plotting codes were sourced from Zhu et al. (14).

#### 2.1.4 Colocalization analysis

We used the coloc R package to perform colocalization analysis to detect shared causal variants between identified immune-related mQTLs, eQTLs, or pQTLs and T2DM. Specifically, when a GWAS signal and QTLs colocalized, we inferred that the GWAS signal might influence the phenotype by altering gene-related biological processes. In the colocalization analysis, five different posterior probabilities corresponding to five independent hypotheses were reported: 1) SNPs are not associated with either trait (H0); 2) SNPs are associated only with gene expression (H1); 3) SNPs are associated only with disease risk (H2); 4) Both traits are associated with SNPs, but with different causal variants (H3); and 5) Both traits share the same causal variant (H4).

According to published literature, the colocalization region windows for mQTL-GWAS, eQTL-GWAS, and pQTL-GWAS colocalization analyses were set to ±500 kb, ± 1000 kb, and ±1000 kb, respectively. The default prior probability for SNPs being associated with both exposure and outcome was set to p12 = 1×10^-5^. While a PPH4 (posterior probability of H4) > 0.8 has been shown to provide strong Bayesian evidence supporting colocalization (16), we initially adopted a relaxed threshold of PPH4 > 0.5 during the exploratory multi-omics phase to maximize screening sensitivity and minimize false negatives for potential downstream *in vivo* targets (17, 18). However, to rigorously ensure methodological robustness, we further conducted sensitivity analyses by applying the conventional, highly stringent double-standard of PPH4 > 0.8 and P_HEIDI > 0.01 (14) to confirm true pleiotropy (a shared causal variant) rather than linkage disequilibrium for our core candidate genes.

### 2.2 Functional validation in a T2DM mouse model

#### 2.2.1 T2DM model induction and intervention

Forty 8-week-old male C57BL/6J mice (25 ± 2 g, SPF grade) were purchased from GemPharmatech Co., Ltd. [license SCXK (Yue) 2023-0067]. After one-week acclimation, the animals were randomly assigned to the normal control group (n = 10) or the model group (n = 30). Control mice received a standard chow diet, whereas model mice were fed a high-fat diet (HFD; 60% kcal fat, 20% protein, 20% carbohydrate) for 12 weeks. Gastroparesis was then induced by oral gavage of activated-charcoal suspension (6.25 g gum arabic, 2.5 g activated charcoal in 50 mL distilled water). Mice were individually housed, and the interval from gavage to the first black stool was recorded; a significant prolongation relative to controls (P < 0.05) confirmed successful DGP. Model mice were subsequently re-randomized into DGP, DGP + retained acupuncture (RA), and DGP + electro-acupuncture (EA) groups (n = 10 each) and maintained on HFD until the end of the study.

For intervention, bilateral Zusanli (ST36) acupoints were selected for needle insertion. The exact anatomical location of ST36 in mice was situated on the posterolateral aspect of the knee joint, approximately 2 mm distal to the fibular head. Following aseptic preparation with 75% ethanol, sterile stainless-steel needles (0.30 mm × 25 mm) were inserted perpendicularly to a depth of 2 ± 0.5 mm into the underlying muscle. RA mice received 1 min manipulation followed by 30 min retention; EA mice received identical needling plus 2 Hz/1 mA pulses (hind-paw twitch) for 30 min; control and DGP mice were restrained for 30 min without needling. Treatments were applied five days per week for seven weeks. Interventions were administered five days per week for seven consecutive weeks. All animal procedures received prior approval from the Animal Ethics Committee of Shenzhen Institute of Advanced Technology (SIAT-IACUC-220914-NS-CZX-A2187) and were conducted in strict accordance with the Guidance for the Care and Use of Laboratory Animals promulgated by the Ministry of Science and Technology of China (2006).

#### 2.2.2 RNA extraction and real-time quantitative PCR

Total RNA was extracted using Trizol according to the manufacturer’s instructions. The purity and concentration of the extracted RNA were determined using NanoDrop 2000. Total RNA was reverse-transcribed into cDNA using the RevertAid First Strand cDNA Synthesis Kit (Thermo), according to the instructions. For qRT-PCR, the samples were mixed with 2x SYBR Green qPCR Master Mix (Selleck), and the mixture was run on a 7900HT Real-Time PCR system. Relative mRNA expression was evaluated using the 2−ΔΔCt method and was normalized to that of GAPDH. The sequences for primers used in this study can be accessed in **Supplementary Table 2**. All qRT-PCR experiments were performed in triplicates.

## 3 Results

### 3.1 Identification of candidate genes via multi-omics analysis

**Figure 2:**
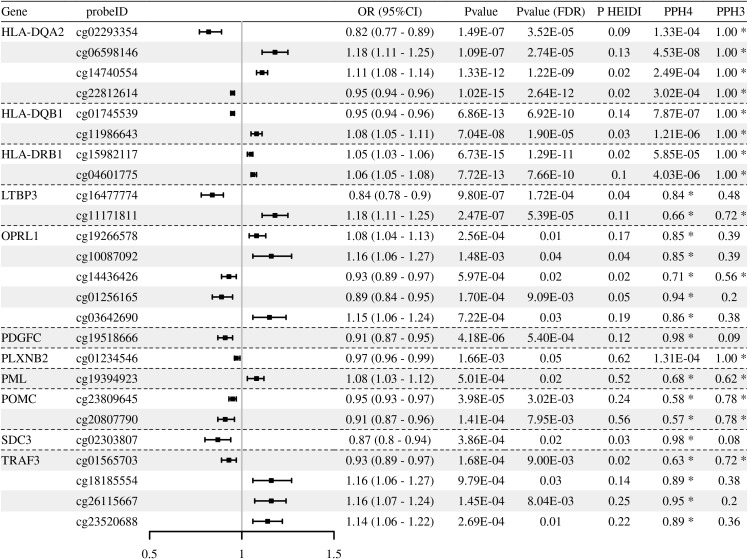
Associations of genetically predicted immune-related gene methylation (mQTLs) with T2DM risk in Mendelian randomization analysis. The forest plot displays the odds ratios (OR) and 95% confidence intervals (CI) for significant CpG sites. Statistical significance was defined by a Benjamini-Hochberg adjusted P-value (P__FDR_) < 0.05 and a HEIDI test P-value (P__HEIDI_) > 0.01. PPH4, posterior probability of Hypothesis 4 in colocalization analysis.

**Figure 3:**
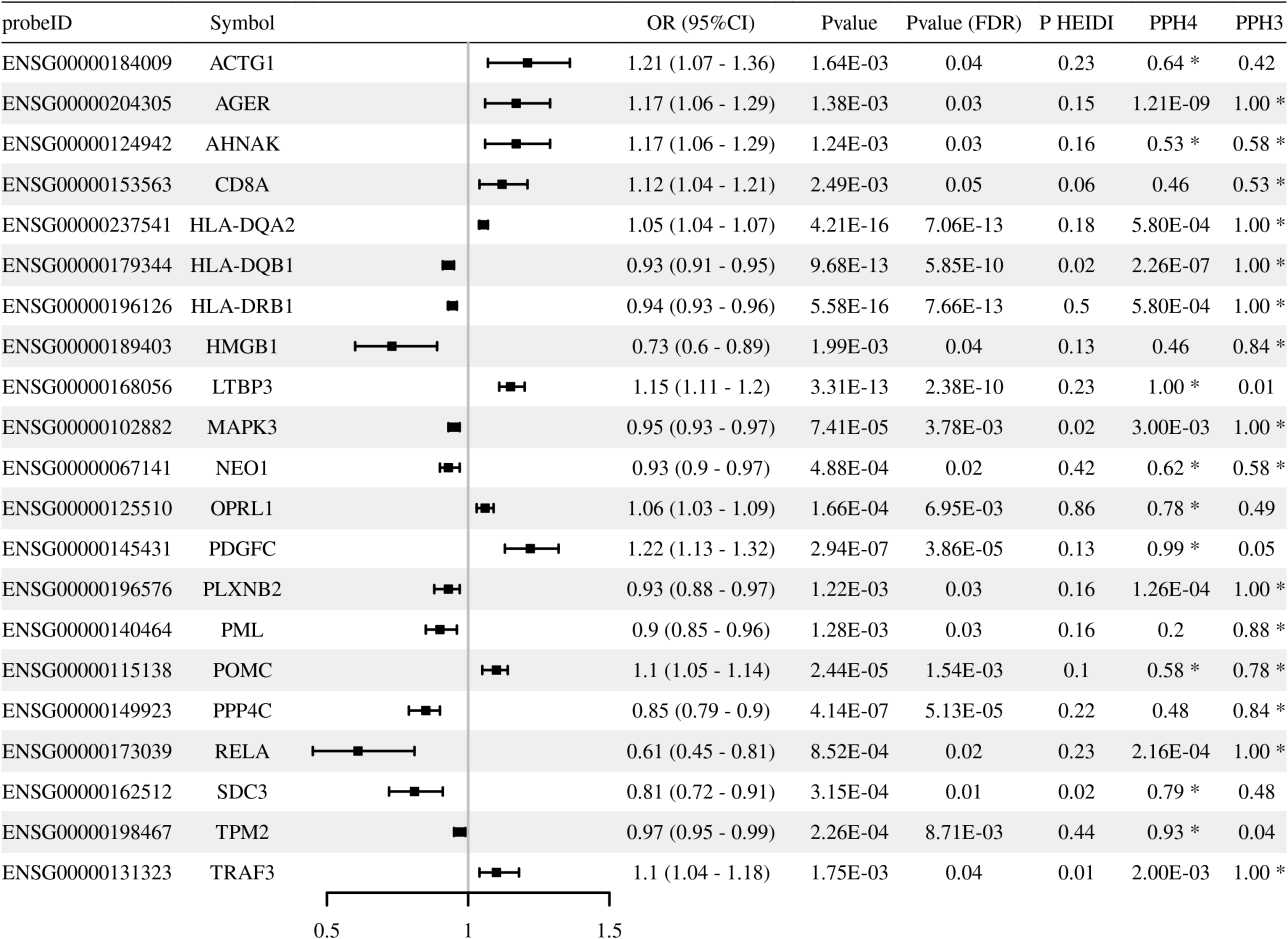
Associations of genetically predicted immune-related gene expression (eQTLs) with T2DM risk in Mendelian randomization analysis. The forest plot displays the odds ratios (OR) and 95% confidence intervals (CI) for significant genes. Statistical significance was defined by P__FDR_ < 0.05 and P__HEIDI_ > 0.01. PPH3, posterior probability of Hypothesis 3.

**Figure 4:**
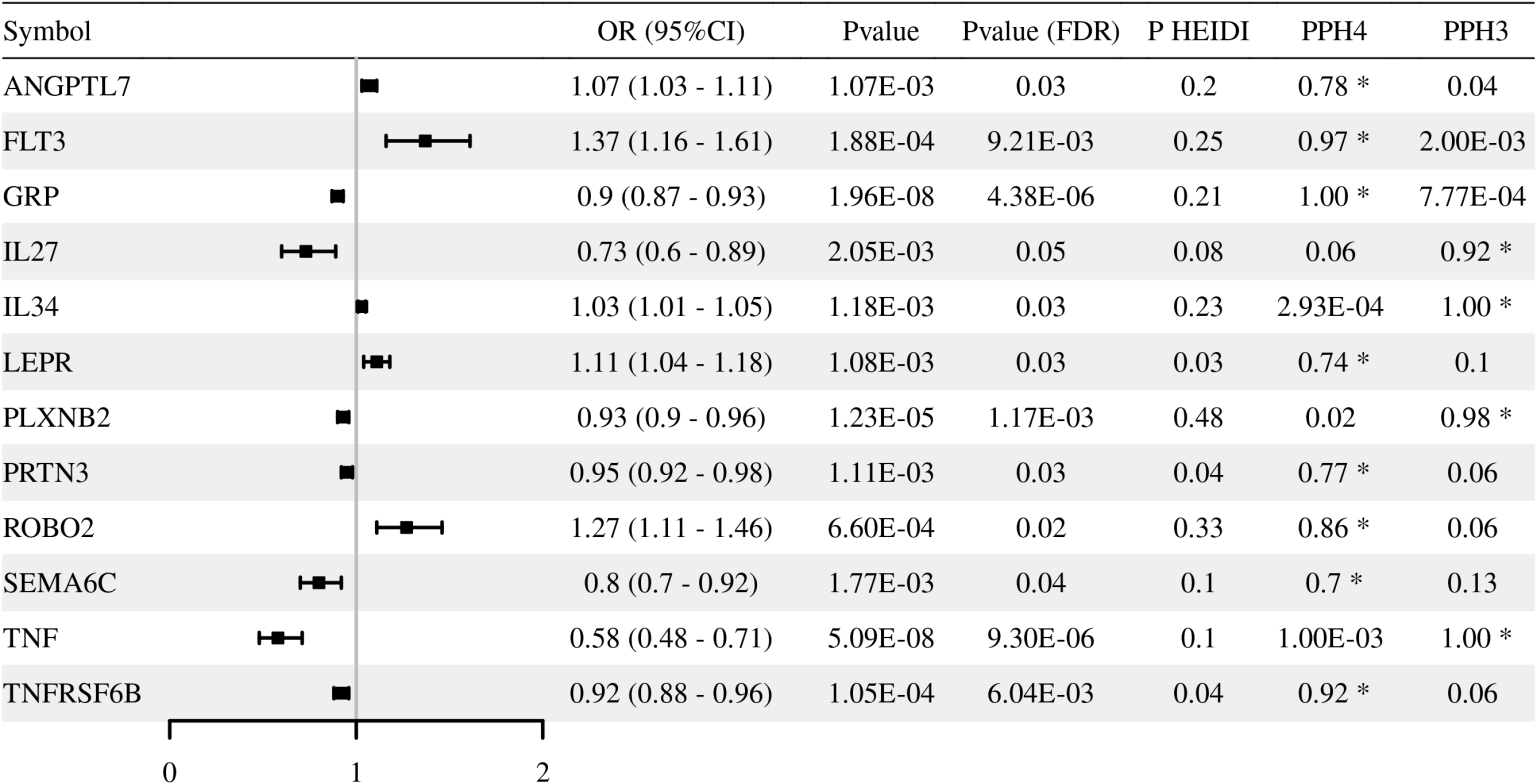
Associations of genetically predicted immune-related protein abundance (pQTLs) with T2DM risk in Mendelian randomization analysis. The forest plot displays the odds ratios (OR) and 95% confidence intervals (CI) for significant proteins. Statistical significance was defined by P__FDR_ < 0.05 and P__HEIDI_ > 0.01.

Through multi-omics analysis, we identified a series of immune-related genes potentially associated with T2DM, including *OPRL1*, *LTBP3*, and *PLXNB2*, which provide targets for further functional validation (**Figures 2**–**4****;****Supplementary Tables 3**-**S8**). Moreover, integration of methylation quantitative trait locis (mQTLs)/expression quantitative trait locis (eQTLs) data revealed that the methylation levels were causally associated with expressions for *LTBP3* (cg16477774), *OPRL1* (cg03642690), and *PLXNB2* (cg01234546) (**Table 1****;****Supplementary Tables 9**, **S10**). Notably, sensitivity analyses demonstrated that the core candidate genes fully withstand rigorous criteria, exhibiting strong colocalization (LTBP3: cg16477774, PPH4 = 0.84; ENSG00000168056, PPH4 = 1.00. OPRL1: cg03642690, PPH4 = 0.86) while concurrently passing the HEIDI test (P_HEIDI > 0.01).

**Table 1:** Results of integrating multi-omics evidence.

Expo ID	Outco Gene	p SMR	p FDR	p HEIDI	OR SMR (95% CI)
cg16477774	LTBP3	2.34E-13	6.69E-12	0.077	0.28 (0.2 - 0.39)
cg03642690	OPRL1	5.09E-10	9.80E-09	0.023	8.81 (4.44 - 17.49)
cg01234546	PLXNB2	1.48E-73	6.11E-71	0.013	1.32 (1.28 - 1.36)
PLXNB2	Plexin-B2	3.90E-119	6.70E-117	1.87E-11	3.28 (2.97 - 3.63)

### 3.2 Tissue-specific validation

We explored causal relationships between identified gene expression and T2DM risk in specific tissues, using pancreatic eQTL data (GTEx V8) to validate blood cis-QTL integration results. *OPRL1* was validated in pancreatic tissue eQTL SMR analysis (**Supplementary Table 11**). Based on these results, *OPRL1* (cg03642690) showed strong colocalization evidence in mQTL, eQTL, and GWAS analyses, further validated in the FinnGen replication set SMR and pancreatic eQTL SMR. Thus, *OPRL1* (cg03642690) may have a causal relationship with T2DM. Evaluating risk via OR values, both cg03642690 methylation (OR = 1.15, 95%CI = 1.06-1.24) and *OPRL1* expression (OR = 1.06, 95%CI = 1.03-1.09) positively correlate with T2DM risk. Additionally, cg03642690 methylation positively correlates with *OPRL1* expression (OR = 8.81, 95%CI = 4.44 - 17.49).

### 3.3 Functional validation of LTBP3 and PLXNB2 in a T2DM mouse model

Based on our multi-omics analysis identifying *LTBP3*, *PLXNB2* and *OPRL1* as key candidate genes, we next sought to validate their functional relevance *in vivo*. The high-fat diet successfully induced a T2DM phenotype in mice, characterized by significant hyperglycemia and reduced glucose tolerance (**Supplementary Figure 1**). Furthermore, T2DM mice exhibited lower small-intestinal transit (P < 0.05), shorter crypts (P < 0.01) and reduced *OCLN* expression (P < 0.05), while gastric emptying and villus length were unchanged (**Supplementary Figure 2**).

**Figure 5:**
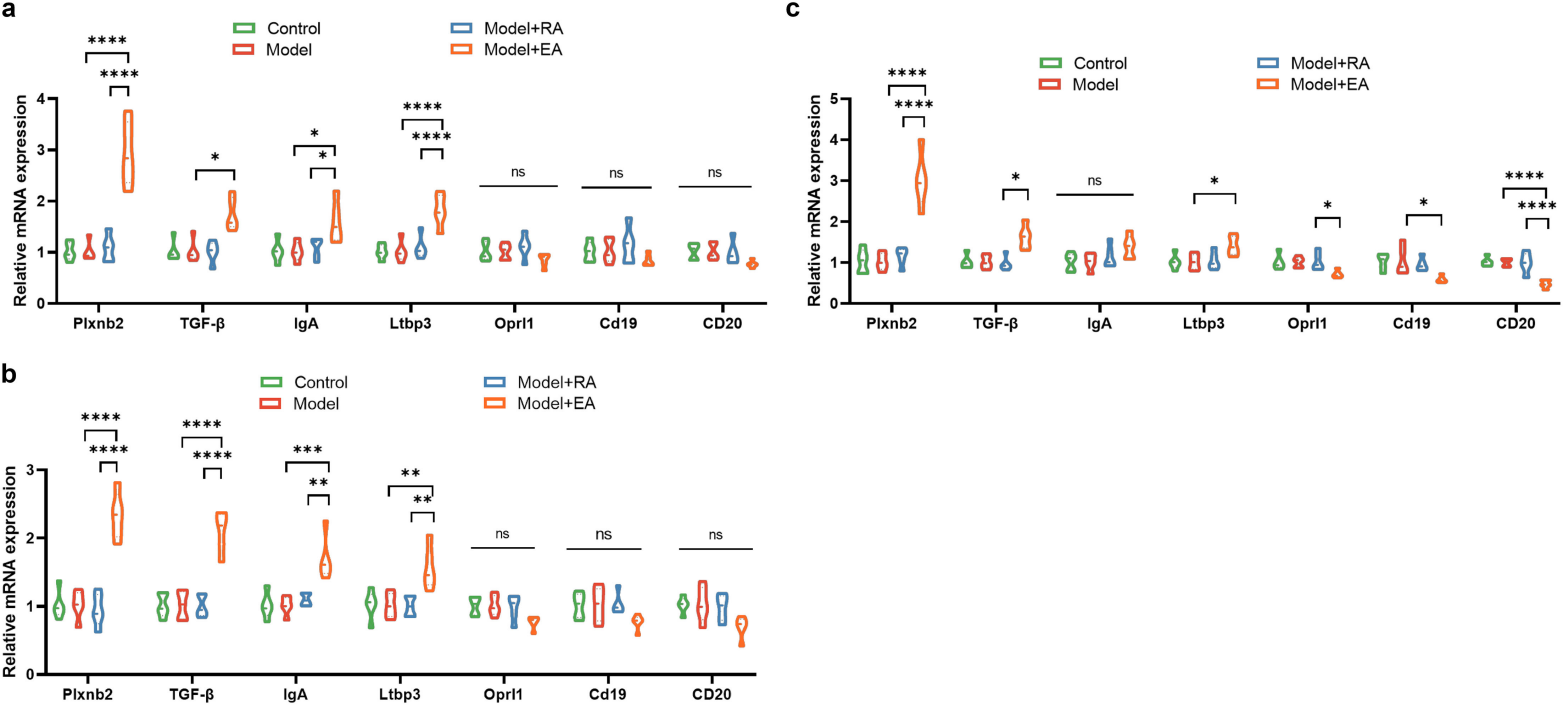
The relative mRNA expression levels of Plxnb2, Ltbp3, Tgfβ, and IgA in the gastrointestinal tissues of mice. Expression levels were quantified using RT-qPCR and normalized to GAPDH. Data are presented as mean ± SD (n = 10 per group). *P < 0.05, **P < 0.01, ****P < 0.0001; ns, not significant. EA, electroacupuncture; DGP, diabetic gastroparesis.

Next, we examined the mRNA expression of *Plxnb2*, *Ltbp3*, *Tgfb1*, and *Igha* in the gastrointestinal tissues of T2DM mice. As shown in **Figure 5**, RT-qPCR analysis revealed that the mRNA expression of both *Ltbp3* and *Plxnb2* was significantly altered in the gastrointestinal tissues of T2DM mice compared to controls (**Figure 5**).

Finally, we assessed the therapeutic effects of EA on these genes. EA at ST36 and ST25 partially restored transit and crypt depth (both P < 0.05), but only modestly elevated *OCLN* (**Supplementary Figure 2**). Thus, EA mitigates diabetic intestinal dysmotility yet has limited impact on overall gastrointestinal function. Notably, EA treatment led to a significant upregulation of *Plxnb2*, *Tgfb1*, *Igha* and *Ltbp3*, suggesting these genes are responsive to therapeutic intervention (**Figure 5**).

## 4 Discussion

This study systematically investigated the causal relationships between the methylation, expression, and protein abundance of immune-related genes and T2DM through multi-omics approaches and *in vivo* validations. While our human genomic analyses identified systemic immune-related risk genes, our *in vivo* validation primarily focused on the gastrointestinal tract using a diabetic gastroparesis (DGP) model. This selection is biologically and clinically well-founded. First, the DGP model is a well-established *in vivo* model for evaluating T2DM and its related target-organ complications (19). Second, systemic immune dysregulation in T2DM is intimately linked to the gut-immune-metabolic axis, and is often profoundly manifested within the gastrointestinal microenvironment (20). Therefore, the DGP model provides a highly relevant pathological window to reliably observe how systemic immune genetic variants interact with the local tissue microenvironment. Consequently, we found that *LTBP3* and *PLXNB2* are key regulators of gastrointestinal immune pathology in T2DM and are responsive to therapeutic interventions.

*PLXNB2* encodes Plexin-B2, regulating immune responses, axon guidance, and cell migration, key in immune and metabolic disorders (21). Recent studies link *PLXNB2* to diabetes pathophysiology (22). *PLXNB2* expression is associated with diabetic neuropathy, and proteomics identified Plexin-B2 as a plasma protein linked to increased T2DM risk via pancreatic dysfunction mechanisms. Our study supports *PLXNB2*’s role, showing negative correlations between T2DM risk and methylation level of cg01234546, *PLXNB2* expression, and Plexin-B2 protein abundance. The positive correlation between cg01234546 methylation and *PLXNB2* expression suggests epigenetic regulation is crucial in controlling *PLXNB2* levels, influencing T2DM-associated metabolic processes. This aligns with findings linking *PLXNB2* expression to impaired glucose homeostasis and T2DM development (23). Importantly, we discovered that T2DM mice exhibited lower small-intestinal transit, shorter crypts, and compromised intestinal barriers, which are key features of gastrointestinal dysfunction. While the exact molecular mechanisms are not fully established, the altered expression of *PLXNB2* may influence intestinal epithelial structure and barrier integrity, contributing to the gastrointestinal symptoms observed in T2DM. In addition, the expression of *PLXNB2* was actively responsive to EA, which positions *PLEXNB2* as a promising candidate for therapeutic modulation. Thus, *PLXNB2* appears significant in the T2DM immune-metabolic network, suggesting it as a potential therapeutic target.

*LTBP3* encodes a protein critical in regulating the *TGF-β* signaling pathway, essential for *TGF-β* secretion, activation, and localization, crucial for processes like proliferation, differentiation, and immune modulation. *LTBP3* is implicated in immune regulation via its role in *TGF-β3* production by Tregs, vital for suppressing excessive immune responses (24). It is also involved in adipose tissue function, promoting brown adipogenesis via *TGF-β2*, impacting energy homeostasis and conditions like obesity and T2DM (25). We provided the first functional evidence in a T2DM mouse model that *Ltbp3* expression is not only associated with the disease state but is also dynamically modulated by EA. This strongly supports *LTBP3* as a viable therapeutic target. *LTBP3* influences gastrointestinal pathology in T2DM by regulating *TGF-β* signaling, which is essential for maintaining intestinal barrier integrity and immune balance (26). Altered *LTBP3* expression can impair barrier function and promote inflammation, contributing to features like shorter crypts and compromised intestinal transit. Therapeutic modulation of *LTBP3* may help restore gut homeostasis in T2DM. Given *LTBP3*’s roles in immune and adipose regulation, alterations in its expression could contribute to T2DM’s inflammatory and metabolic dysregulation.

*TGF-β* is a multifunctional cytokine involved in immune response, cell proliferation, and tissue remodeling. In T2DM, elevated *TGF-β* levels are associated with inflammation, fibrosis, and complications like diabetic nephropathy (27). High glucose levels in T2DM upregulate TGF-β expression and activate its signaling pathways, thereby promoting disease progression and tissue damage (27). On the other hand, IgA is crucial for mucosal immunity and maintaining intestinal barrier function. Changes in intestinal IgA levels are linked to metabolic dysregulation in T2DM and can affect glucose homeostasis (28). Our findings indicated that the expression levels of *Tgfb1* and *Igha* were elevated in the gastrointestinal tissues of T2DM model mice. Moreover, EA treatment further increased their expression in these models. Rather than exacerbating the pathological state, this targeted upregulation by EA likely reflects an enhanced compensatory response to promote mucosal tissue repair (via *TGF-β*) and reinforce the compromised intestinal immune barrier (via *IgA*). This suggests a close relationship between immunity and the pathophysiology of T2DM.

In this study, diabetic gastroparesis (DGP) mice display segmental gut failure, including slowed transit, crypt loss, barrier leak. EA at ST36/ST25 partly rescues transit and crypt depth, but OCLN remains low, indicating residual dysfunction. SMR analyses have converged on two matricellular proteins, *LTBP3* and *PLEXNB2*, as novel regulators of diabetic complications. Given their altered expression in our DGP model, *LTBP3* and *PLEXNB2* may collectively contribute to the structural and functional gastrointestinal deficits observed. EA appears to partially reverse these pathological changes, highlighting their potential as therapeutic targets. However, the precise molecular mechanisms mediating these effects require further experimental validation.

Additionally, in single-level mQTL/eQTL validation, methylation of CpG site cg19518666 within *PDGFC* and the gene’s expression were validated via colocalization and replication datasets. *PDGFC* encodes a protein vital for cell growth, proliferation, and angiogenesis, implicated in metabolic and cardiovascular diseases. *PDGFC* promotes angiogenesis and interacts with *PDGFRα* (29). In T2DM, *PDGFC* is linked to regulating insulin resistance and glucose metabolism (30). Our study supports this, finding cg19518666 methylation negatively correlates with T2DM risk, while *PDGFC* expression positively correlates. This suggests lower methylation might increase *PDGFC* expression, potentially contributing to T2DM pathogenesis by promoting inflammation and fibrosis, exacerbating insulin resistance and β-cell dysfunction (31). These findings underscore *PDGFC* as a significant regulator in the T2DM-associated immune-metabolic network.

Our SMR analysis revealed that hypermethylation at the OPRL1 CpG site cg03642690 is positively associated with both increased OPRL1 expression and higher T2DM risk. Specifically, the notably large effect size observed (OR = 8.81) biologically reflects the robust cis-regulatory impact of this CpG site on local gene transcription. As previously established in multi-omics SMR frameworks, such critical methylation loci can act as potent epigenetic switches, leading to exponentially amplified downstream transcriptional responses (32, 33). Given that the OPRL1-encoded NOP receptor modulates immune cell activity, this amplified epigenetic upregulation may exacerbate T2DM-associated chronic inflammation and metabolic imbalances (34, 35). However, in our *in vivo* validation, Oprl1 expression was non-responsive to T2DM and EA interventions in intestinal tissues. This discrepancy likely stems from tissue-specific expression differences, as our *OPRL1* SMR findings are primarily driven by whole-blood QTL data. Recent studies support that the NOP receptor system predominantly acts as a systemic peripheral immune factor driving macrophage recruitment, rather than a constantly expressed functional factor in the local gut microenvironment (36, 37). Therefore, its lack of functional confirmation in our local DGP model underscores the tissue-specific nature of immune regulation, warranting further research into its role as a systemic biomarker.

In this study, our multi-omics SMR approach successfully constructed a multi-dimensional regulatory network linking immune-related genes to T2DM. However, several limitations must be acknowledged. First, despite the integration of multi-omics data, the analysis remains observational, meaning these preliminary associations require further validation in independent population cohorts to ensure generalizability. Specifically, the GWAS and QTL datasets utilized are predominantly derived from populations of European ancestry, which may limit the applicability of our findings across diverse ethnic groups. Second, our reliance on summary-level data precludes the ability to perform stratified analyses or to evaluate potential non-linear causal relationships. Third, because our SMR approach inherently relies on cis-QTLs from localized gene regions rather than multiple independent genome-wide variants, traditional multi-instrument sensitivity analyses (such as MR-Egger or weighted median) cannot be applied. While we rigorously applied the HEIDI test to minimize linkage bias, the possibility of residual or unmeasured horizontal pleiotropy cannot be entirely ruled out in Mendelian randomization frameworks. Most importantly, a translational mismatch exists between the systemic T2DM risk reflected in the human GWAS data and our *in vivo* validation, which was primarily restricted to gastrointestinal tissues in the DGP mouse model. This tissue-specific discrepancy explains why systemically highlighted genes like *OPRL1* were non-responsive in our local gut samples. To address these gaps and avoid overstating mechanistic claims, future functional studies must comprehensively validate these key targets (e.g., *LTBP3*, *PLXNB2*, and systemically acting *OPRL1*) in classical metabolic tissues—such as the pancreas, liver, and adipose tissue—as well as in systemic immune cells from human clinical samples. This will fully elucidate their roles in systemic T2DM pathogenesis and clarify the precise molecular mechanisms underlying EA interventions.

## 5 Conclusions

This study preliminarily explored the potential roles of immune-related genes in T2DM through SMR analysis and multi-omics approaches, while also discussing the possible mechanisms of *OPRL1* in immune processes and T2DM, as well as the roles of *PLXNB2*, *PDGFC*, and *LTBP3* in this context based on existing literature. These findings not only highlight the complex role of epigenetic regulation in disease progression but also suggest that future research should focus on uncovering the intricate interactions among these genes to comprehensively elucidate the intertwined pathophysiological mechanisms of immune processes and T2DM. Ultimately, this may provide more precise targets for disease prevention and treatment.

## Data Availability

The original contributions presented in the study are included in the article/**Supplementary Material**. Further inquiries can be directed to the corresponding author.
