# Supplementary material for: Multi-omics identifies a T2DM-associated immune-regulatory network modulated by electroacupuncture
Source: Front Endocrinol (Lausanne). 2026 Apr 1;17:1742131. doi: 10.3389/fendo.2026.1742131 (PMC13079183; doi:10.3389/fendo.2026.1742131)
Supplement: Supplementary file 1 [file DataSheet1.docx]

**Supplementary materials**


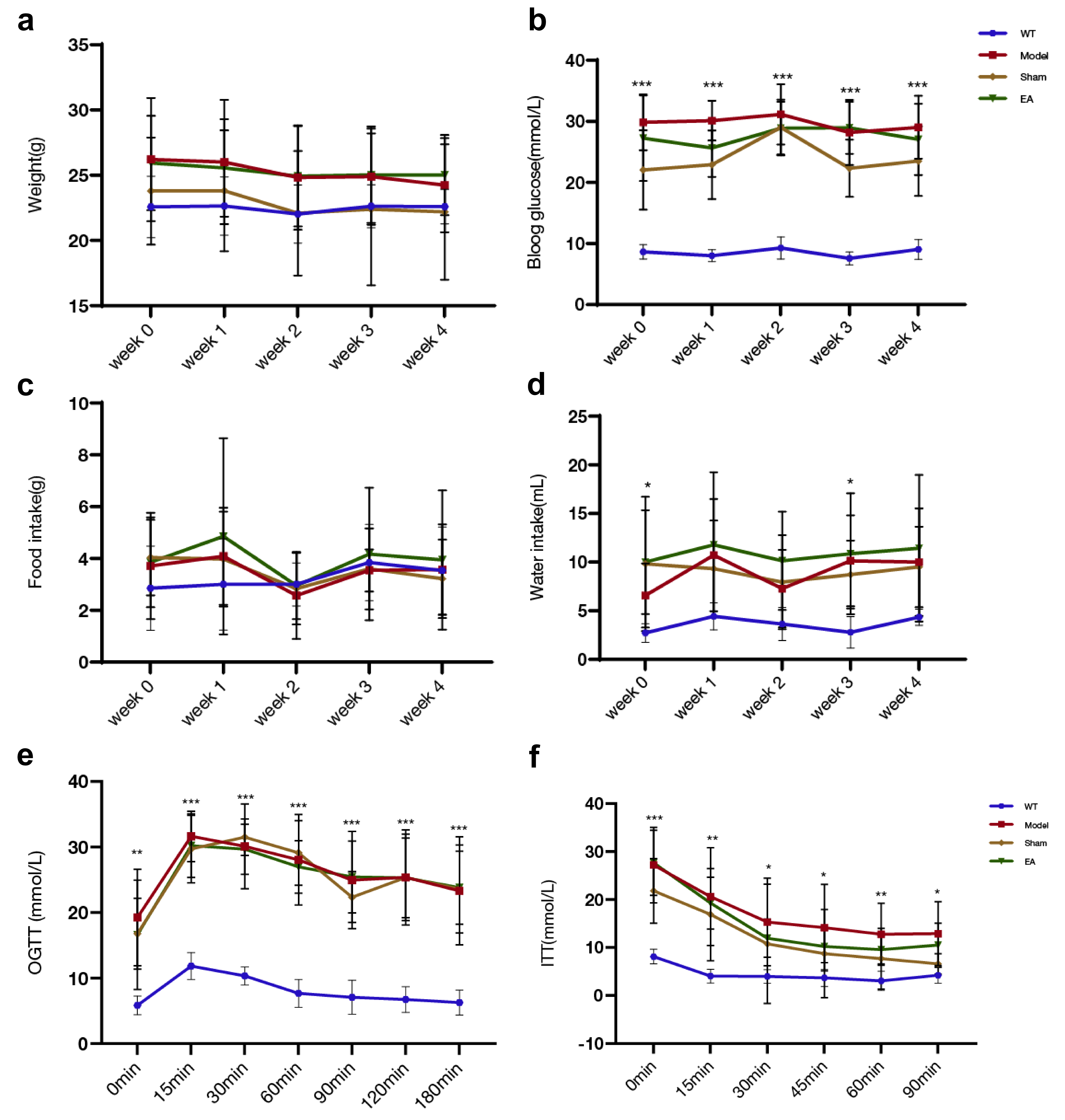


**Supplementary Figure S1.** Biochemical characteristics of T2DM mice. (a) Body weight, (b) fasting blood glucose, (c) daily food intake, (d) water intake, (e) oral glucose tolerance and (f) insulin tolerance in mice.


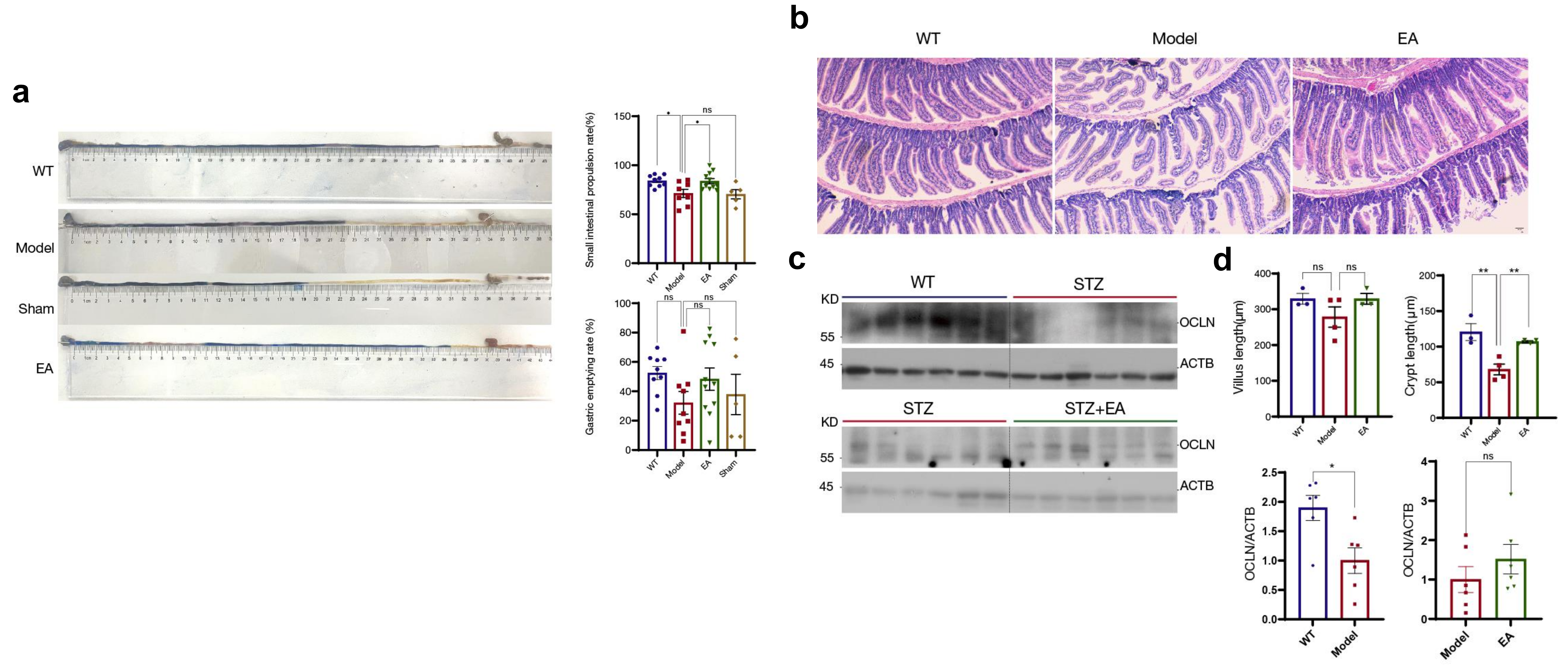


**Supplementary Figure S2.** Determination of small-intestinal propulsion rates, gastric emptying and assessment of small-intestinal barrier function in mice. (a) Small-intestinal propulsion rates and gastric emptying rates were compared among different groups of mice. (b) Representative H&E images showing crypt morphology. (c) Relative protein expression of OCLN in different groups of mice. (d) Statistical analysis of villus length, crypt length and OCLN expression.
